# Effects of increasing drip irrigation at different maize growth stages on soil microorganisms

**DOI:** 10.3389/fmicb.2024.1343302

**Published:** 2024-01-31

**Authors:** Lei Wang, Xiaojuan Wang, Tianle Wang

**Affiliations:** ^1^Shanxi Institute of Organic Dryland Farming, Shanxi Agricultural University, Taiyuan, Shanxi, China; ^2^College of Agriculture, Shanxi Agricultural University, Taiyuan, Shanxi, China; ^3^State Key Laboratory of Integrative Sustainable Dryland Agriculture (In Preparation), Shanxi Agricultural University, Taiyuan, Shanxi, China; ^4^Key Laboratory of Sustainable Dryland Agriculture (Co-construction by Ministry of Agriculture and Rural Affairs and Shanxi Province), Shanxi Agricultural University, Taiyuan, Shanxi, China; ^5^Shanxi Province Key Laboratory of Sustainable Dryland Agriculture, Shanxi Agricultural University, Taiyuan, Shanxi, China

**Keywords:** maize, drip irrigation, soil bacteria, soil fungus, community structure, gene functional prediction, PCA analysis

## Abstract

**Introduction:**

To investigate the effects of different drip irrigation periods on soil microbial communities and functions.

**Methods:**

Increasing drip irrigation amount at the seedling (S), jointing (J), bell (B), tasseling (T) and grain filling (G) stages of maize were studied using no increase in irrigation amount as control (CK). Principal component analysis was conducted to comprehensively evaluate soil microbial quality following the different drip irrigation treatments. In addition, the characteristics of the community structure and the potential functional composition of soil bacteria and fungi were comparatively analyzed by combining amplicon sequencing and functional prediction methods.

**Results:**

The results indicated that MBNT15 was the most important genus for the classification of soil bacterial samples, Saitozyma was the most important genus for the classification of soil fungal samples, and fungi were more important than bacteria for the classification of soil microbial samples. Compared with fungal communities, bacterial communities exhibited high levels of functional diversity. The proportion of metabolism was the highest in the prediction of bacterial primary functions, and carbohydrate metabolism and amino acid metabolism were important functions in the prediction of bacterial secondary functions. BugBase phenotype prediction results showed that soil bacteria under B treatment had a higher number of aerobic bacteria and greater resistance to disease and stress. The J treatment had the highest number of bacteria with biofilm forms, and the J, S, and G treatments contained more potentially pathogenic bacteria but fewer stress-tolerant bacteria compared with the CK treatment. The number of Saprotroph was the largest and the number of Symbiotroph was the least. The relative abundances of Saprotroph, Pathotroph and Symbiotroph were 68.60%~74.33%, 15.76%~20.60% and 9.16%~11.13%, respectively.

**Discussion:**

The findings provide a reference for conserving water resources, improving maize yield, and predicting soil microbial metabolic potential and function by reflecting the richness of the soil microbial community structure in maize fields.

## 1 Introduction

China is a country with serious drought and water scarcity. The problem of water resource shortage is currently one of the most concerned focuses. In the use of water resources, water for agricultural irrigation is the main type of water consumption. According to statistics, the total water consumption of the whole country was 602.12 billion m^3^, and the water consumption for agriculture was 368.23 billion m^3^, which accounted for 61.2% of the total water consumption (Ministry of Water Resources of the People's Republic of China, 2018). Thus in order to develop agriculture, the first thing to do is to develop water-saving and irrigation agriculture, utilizing limited water resources to create greater economic value in agriculture. Common water-saving irrigation methods include border irrigation, furrow irrigation, flood irrigation, drip irrigation, sprinkler irrigation, micro irrigation, infiltration irrigation, and mulching irrigation (Wang, 2020). Drip irrigation has the advantages of conserving and rationally distributing water resources, improving crop water and fertilizer use, and increasing crop yield and quality compared with other water-saving irrigation methods (Sun et al., 2020). Shanxi Province is located in the arid and semi-arid region of the Loess Plateau, with limited arable land resources, maize as one of the main grain crops, the sown area has been increasing year by year, and it is the largest food crop in terms of area under cultivation (Li et al., 2022). Therefore, it is crucial to use water-saving drip irrigation technology to promote the development of maize plantation industry and local economic development.

Soil is the environment for plant growth and development, in addition to the nutrients and soil air necessary for plant growth and development, there are also a variety of microorganisms in large numbers, these soil microbial communities in terrestrial ecosystems of almost all the biogeochemical cycling processes play a key role (Li et al., 2023). Microorganisms affect crop production directly or indirectly by promoting soil organic matter decomposition and inhibiting the growth of pathogenic bacteria. Different methods of drip irrigation result in different soil environments, soil nutrient status affects the diversity and community composition of soil microorganisms (Lauber et al., 2008), which in turn affects crop yield and quality.

Xu et al. (2015) found that the application of drip irrigation during the maize seedling stage could effectively alleviate the effects of water shortage. Zhu et al. (2023) found that the soil microbes in cereal fields were affluent in structure and function. By comparing the rhizospheric soil microorganisms in transgenic insect-resistant maize HGK60 and ordinary maize Zheng 58 fields, Chen et al. (2021) showed that the growth stage was one of the factors affecting rhizospheric soil microorganisms. Continuous cropping can harm plant growth. Studies have shown that in tomato soils with continuous cropping, the dominant soil microbial populations shifted from a bacterial-dominated state to a fungal-dominated one. This shift led to a gradual decrease in stability and functional diversity (Zhou and Yang, 2023). The hypothesis was that the number of soil bacteria and fungi was more stable and the function was more abundant. Although the studies on soil microorganisms planting corn have made great progress, the ecological environment and cultivation factors in arid and semi-arid areas of China are constantly changing. Therefore, it is particularly important to explore the growth stage when corn can improve the structure of soil microbial communities as much as possible, in order to improve the soil environment and realize sustainable development.

## 2 Materials and methods

### 2.1 Site description

The experiment was conducted in a dry shed (112°40′5″E, 37°33′22″N) at the Dongyang Experimental Demonstration Base of Shanxi Academy of Agricultural Sciences, Shanxi Agricultural University, which is located in Dongyang Village, Dongyang Town, Yuci District, Jinzhong City, Shanxi Province, and has a temperate continental semi-arid monsoon climate, with an altitude of 802 m above sea level, flat terrain, the average annual precipitation is 410–490 mm, most of which is concentrated in April–September, with an average annual air temperature of 9–10°C, a large difference in temperature between day and night, active cumulative temperature of about 3,990°C (≥0°C), the annual sunshine hours is 2,535–2,662 h, and a frost-free period of about 158 d. The deep groundwater at this experimental site had no effect on the experiment, and 322 mm of rain fell during the maize reproductive stage in the experimental year.

### 2.2 Materials for experimentation

The test maize (*Zea mays* L.) variety was “Dafeng 30,” which was provided by Shanxi Dafeng Seed Industry Co., Ltd.

### 2.3 Experimental design

The experiment was arranged in randomized blocks with single factor control treatment to increase irrigation for different growth stages of maize. There were six treatments (Table 1). The control group (CK) was watered 72 mm in each growth stage. During the entire growth period of maize, CK watered 360 mm. 96 mm of drip irrigation was increased at the seedling stage (S), the jointing stage (J), the large bell stage (B), the tasseling stage (T) and the grain filling stage (G), respectively. S, J, B, T, and G treatments watered 456 mm during the whole growth period of maize. Three replications for each treatment, and a plot area of 25 m^2^, the intervals were filled with cement as an isolation belt. To avoid being affected by natural rainfall, the maize was planted in automatic rain shelters, the water source of maize whole growth stage is all from drip irrigation. The drip irrigation belts were placed 8–10 cm from the maize rootstock and watered 10 days per week. Before planting, each plot received an even distribution of basal fertilizer consisting of *N* 150 kg/hm^2^ and P_2_O_5_ 60 kg/hm^2^. The ground was rotary plowed to a depth of 10 cm using a small type rotocultivator. Afterwards, seeds were manually sown at a rate of 60,000 plants per hectare. This experiment was fertilized and prepped on May 2nd, planted on May 13th, and harvested on September 30th.

**Table 1:** Processing settings for the experiment.

Treatment	Drip irrigation amount (mm)					
	Seedling stage	Jointing stage	Large bell stage	Tasseling stage	Grain filling stage	Whole growth stage
CK	72	72	72	72	72	360
S	168	72	72	72	72	456
J	72	168	72	72	72	456
B	72	72	168	72	72	456
T	72	72	72	168	72	456
G	72	72	72	72	168	456

S, J, B, T, and G treatments mean increasing drip irrigation by 96 mm during the seedling stage, the jointing stage, the large bell stage and the tasseling stage and the grain filling stage, respectively.

### 2.4 Experimental design and treatments

After crop harvest, in October 2021, 18 samples were collected using a small soil earth drill with the five-point sampling method for 0–20 cm of soil. Samples were thoroughly mixed, stones, roots, and other contaminants were removed from the samples, and using a 2 mm aperture soil sieve to remove bulk soil. The soil samples were put into sealed pockets and temporarily preserved with dry ice before being deposited into an ultra-low temperature freezer set at −80°C. This method ensures sufficient backup for measuring the number, diversity, and community structure of soil bacteria and fungi, as well as predicting their gene function. Nucleic acid extraction was performed using the TGuide S96 Magnetic Bead Method Genomic DNA Extraction Kit designed for soil samples. After extracting the DNA of the samples and sequenced. Bacteria and fungi were sequenced separately using Illumina HiSeq and Illumina Novaseq high-throughput sequencing platforms, respectively. The original data underwent quality filtering using Trimmomatic, identification and removal of primer sequences by Cutadapt, splicing of bipartite sequences, and removal of chimeras using USEARCH. The original sequences were then quality-controlled to obtain high-quality sequences, which were used to obtain the final sequences, and the high-quality sequences were clustered and then divided into OTUs, and the sampling effective sequences had basically covered all species in the treatments, which could truly and uniquely respond to the composition of soil microbial communities in each treatment. The taxonomic significance of soil microorganisms was analyzed using random forest and PCA analyses to determine the composition of soil microbial samples. Finally, diversity and differentiation analyses were performed to reveal the functional characteristics of the samples in terms of soil microbial communities distribution structure and growth and development characteristics.

### 2.5 Data processing and calculation methods

Excel 2016 was utilized for initial data processing, Illumina HiSeq and Illumina NovaSeq high-throughput sequencing platforms were utilized. Data processing was accomplished using Trimmomatic (version 0.33), while PICRUSt2 and FUNGuild were utilized for functional gene prediction. Statistical analysis was performed using Excel 2003 and SPSS 18.0. The R language tool was utilized to plot PCA.

## 3 Results

### 3.1 Soil microbial random forest analysis

**Figure 1:**
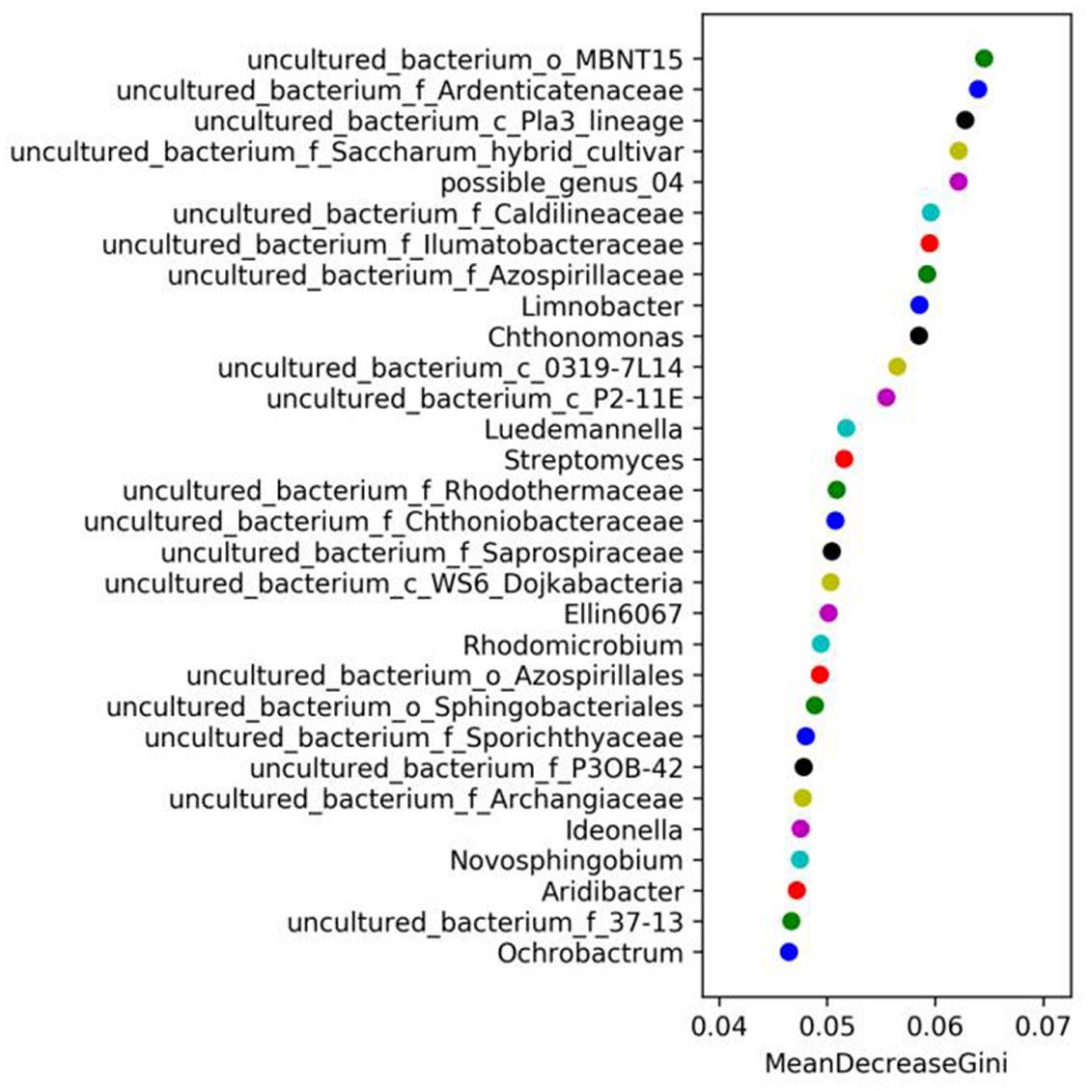
Bacterial random forest analysis diagram under different treatments.

**Figure 2:**
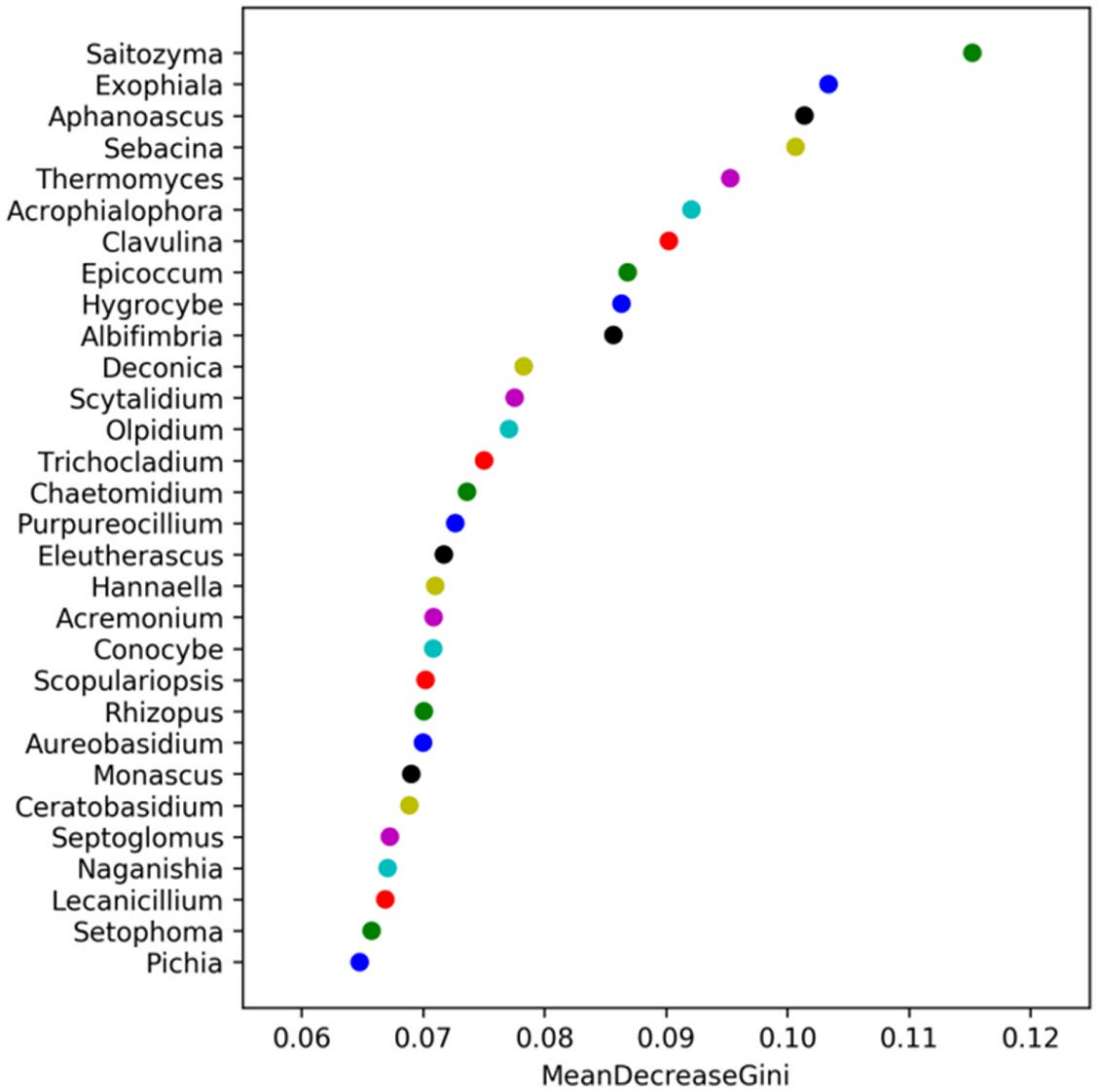
Fungal random forest analysis under different treatments.

Random forest analysis employs multiple decision trees for sample classification and identifying characteristic species, with the importance of the differences between samples having a significant impact on random forest analysis graph of the horizontal coordinate shows the average reduction of the Gini coefficient, the higher the number, indicating that the species of the sample classification of the importance of the species is higher. After analyzing various drip irrigation treatments on maize (Figure 1), the soil uncultured bacteria MBNT15, Ardenticatenaceae, and lineage displayed the highest mean reduction in Gini coefficients. Consequently, they were deemed the most critical for sample categorization. In order of importance for sample classification, Limnobacter, Luedemannella, Rhodomicrobium, Ideonella, Novosphingobium, and Aridibacter displayed a progressively lower mean reduction in Gini coefficients, and Ochrobactrum was the least critical for sample classification. Saitozyma, Exophiala, and Aphanoascus exhibited the highest average reduction of the Gini coefficients and had the highest importance for the classification of the soil fungi samples after different drip irrigation treatments, the importance of Sebacina, Thermomyces, Acrophialophora, Clavulina, Epicoccum, and Hygrocybe to sample classification decreased in turn, whereas Pichia exhibited the lowest average reduction of the Gini coefficients and had the lowest importance for sample classification of the soil fungi in the soil (Figure 2). In the classification of microorganisms in the soil, it was found that fungi had a higher average reduction of Gini coefficients than bacteria, according to the values of the horizontal coordinates in the following two graphs.

### 3.2 PCA analysis of soil microorganisms

**Figure 3:**
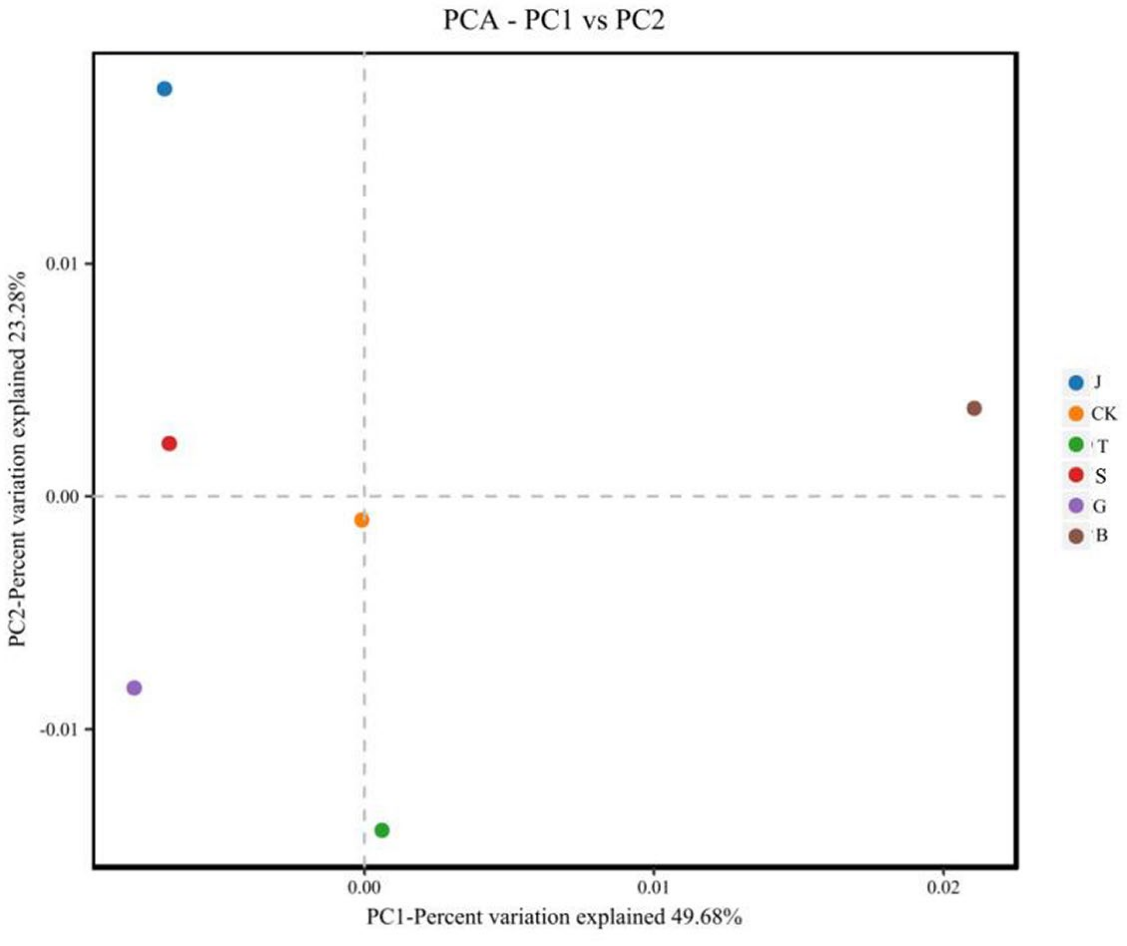
PCA analysis of bacteria under different treatments.

**Figure 4:**
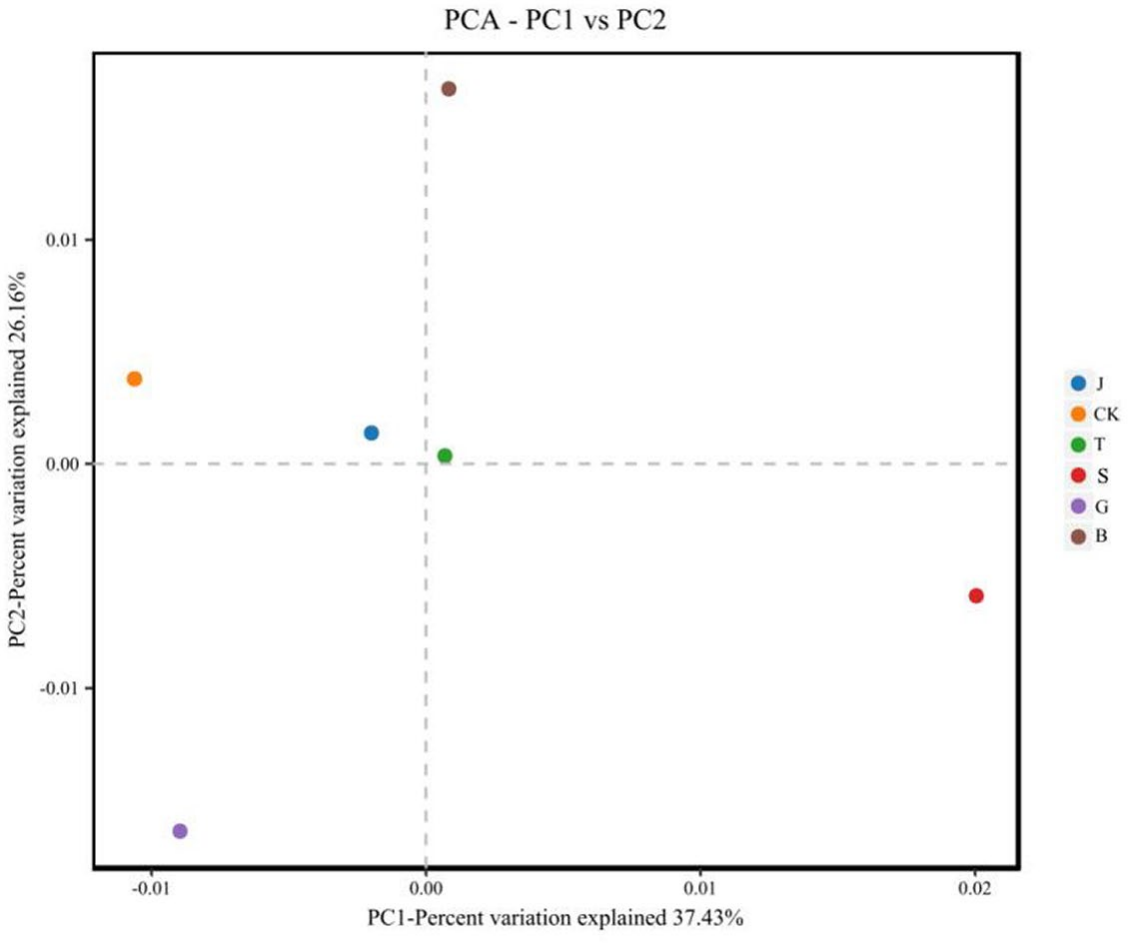
PCA analysis of fungi under different treatments.

Principal Component Analysis (PCA) is a method of analyzing and simplifying data sets by breaking down the variance and illustrating the distinctions between multiple data sets on a two-dimensional coordinate plot (Dubois et al., 2010). The closer the distance between two samples, the more alike their compositions. As shown in Figure 3, following various drip irrigation treatments, soil bacteria made up 49.68 and 23.28% of the variations in the first and second principal components, respectively. The combined contribution of these two components was 72.96%, satisfying the criteria for information extraction. Additionally, the B treatment had a notably high positive loading under the first principal component. The B treatment and the first principal component showed a clear correlation, while the J, S, and B treatments displayed positive loadings for the variable factors in their second principal components. The J treatment had the highest and strongest control in the second principal component. Furthermore, the differences in the principal component composition of the B treatment were most evident for the soil’s bacteria when compared to the CK treatment, whereas the differences were most similar for the J treatment. Soil fungi, as shown in Figure 4, are responsible for the first principal component with contribution values of 37.43 and 26% for the second principal component. Soil fungi, as shown in Figure 4, are responsible for the first principal component with contribution values of 37.43 and 26% for the second principal component. The first principal component exhibits positive loadings for the S, B, and T treatment variable factors, with the J treatment having the highest correlation. The second principal component reveals higher positive loadings for the B treatment variable factor, which is also the most strongly influenced by the control. Compared with CK treatment, the fungal S treatment results in the most significant differences in the principal components, while the J treatment shows the most similarities in differences. Therefore, these two components are responsive to most of the data.

### 3.3 Prediction of PICRUSt2 function of soil bacteria

**Figure 5:**
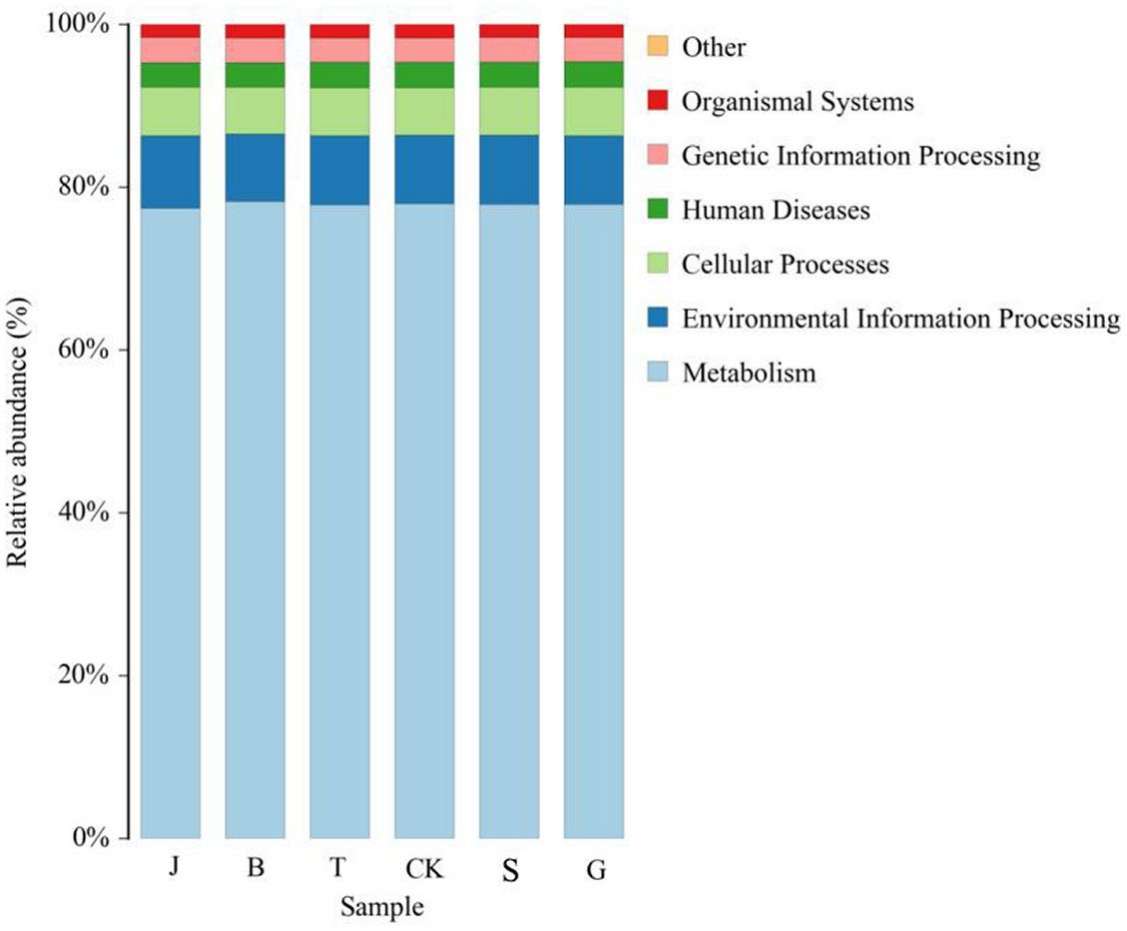
Spectral relative abundance of PICRUSt2-based predicted function for bacteria (hierarchy level 1).

Based on PICRUSt2 analysis to predict the functions of bacterial communities (Figure 5), bacteria in the soil primary function was Metabolism in response to drip irrigation treatments during different reproductive stages of maize, and this function was strongest in the soil of the T, with the relative abundance of this function accounting for 78.24%; and the increase of this function was weakest in the soil of the B, with the relative abundance of this function accounting for 77.45%. Additionally, the following functions are present: Environmental Information Processing, Cellular Processes, Human Diseases, Genetic Information Processing, and Organismal Systems. Moreover, the relative abundance of bacteria with these functions is declining. Differences were significant in the functioning of the B and J with increased drip irrigation for the treatment of Human Diseases, in the G for Genetic Information Processing and the J for the functioning of Organismal systems compared to the control treatment CK (Table 2).

**Table 2:** The main primary functional type index of bacterial PICRUSt2.

Treatment	Metabolism	Environmental information processing	Cellular processes	Human diseases	Genetic information processing	Organismal systems
T	0.779a	0.085a	0.059a	0.031a	0.030ab	0.016ab
G	0.779a	0.085a	0.060a	0.032a	0.030b	0.016ab
B	0.782a	0.083a	0.057a	0.030b	0.031ab	0.016a
S	0.779a	0.085a	0.059a	0.031ab	0.030ab	0.016ab
CK	0.780a	0.084a	0.059a	0.031ab	0.030ab	0.016ab
J	0.775a	0.089a	0.060a	0.030b	0.031a	0.016b

In the same column different small letters refer to difference among treatments at *p* < 0.05 level. S, J, B, T, and G treatments mean increasing drip irrigation by 96 mm during the seedling stage, the jointing stage, the large bell stage and the tasseling stage and the grain filling stage, respectively.

**Figure 6:**
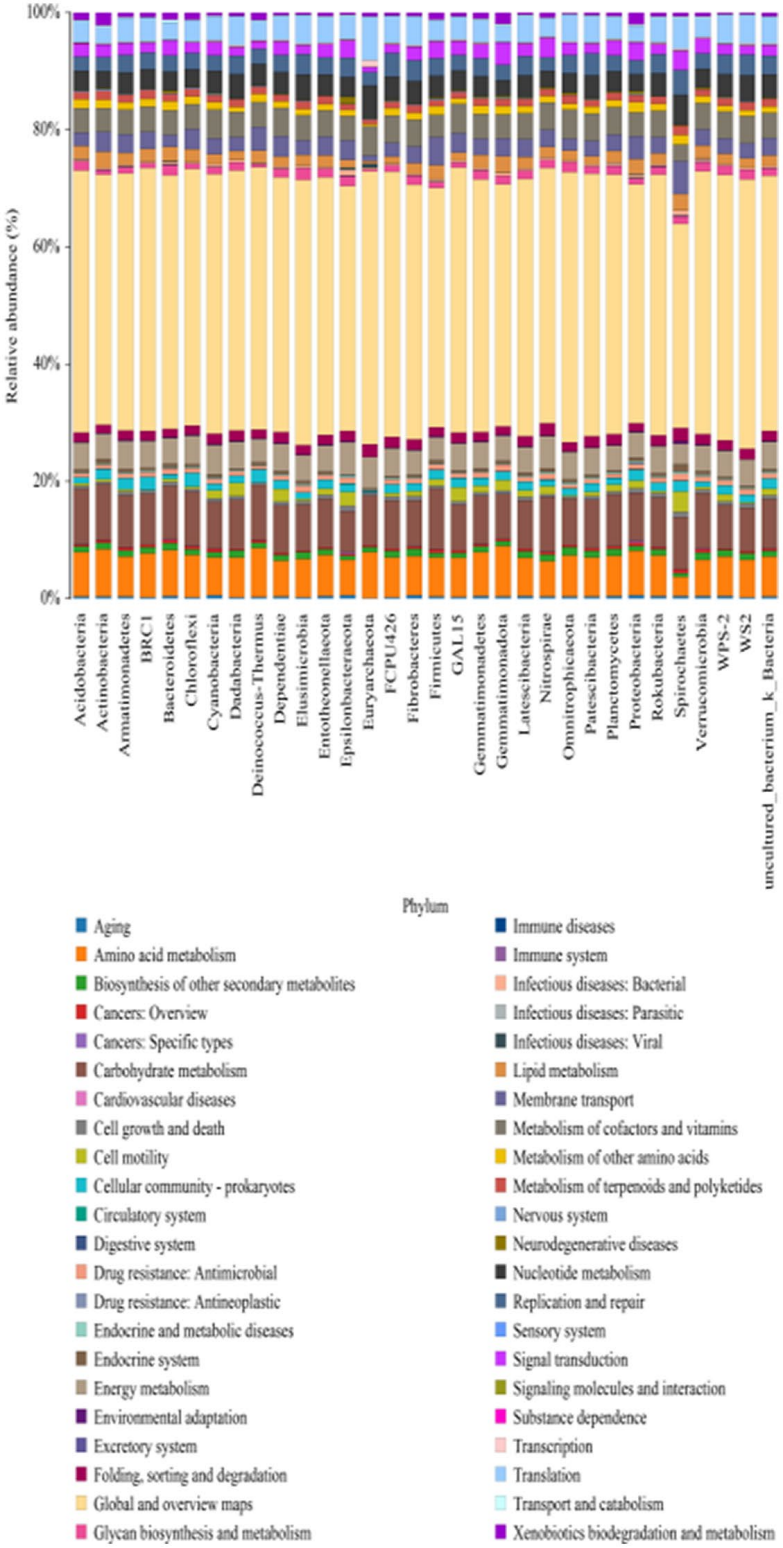
Prediction of bacterial KEGG function under different treatments (hierarchy level 2).

**Figure 7:**
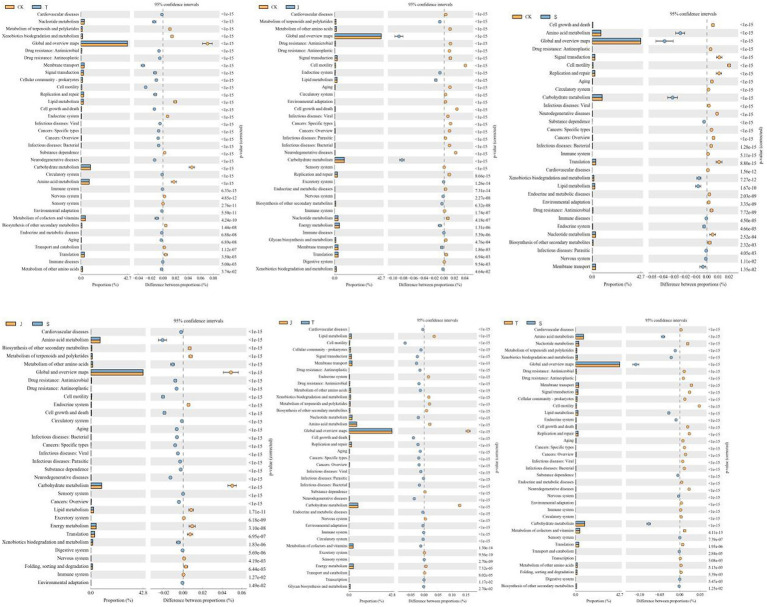
Analysis of KEGG metabolic pathway difference in bacteria under different treatments.

The analysis of the predicted genes at the secondary functional level using the KEGG database identified 44 main pathways (Figure 6). The main functions observed were Global and overview maps, with a richness of 42.58 –42.75%, followed by Carbohydrate metabolism at 8.76–8.87%, and Amino acid metabolism at 7.60–7.62%. Furthermore, there are additional functions such as Biosynthesis of other secondary metabolites, Cardiovascular diseases, Cell growth and death, Cell motility, Cellular community-prokaryotes, Endocrine system, Excretory system, and more. Significant differences in Carbohydrate metabolism and Amino acid metabolism functions were observed between the J and the S. The relative abundance share of the Carbohydrate metabolism function was higher in the J than in the S, while the relative abundance share of the Amino acid metabolism function was the opposite. Global and overview maps functions also differed significantly in the J, T, and S treatments compared to the control treatment CK. The relative abundance share of this function was in the following descending order: J > S > CK > G > T > B (Figure 7).

### 3.4 Prediction of BugBase phenotype of soil bacteria

**Figure 8:**
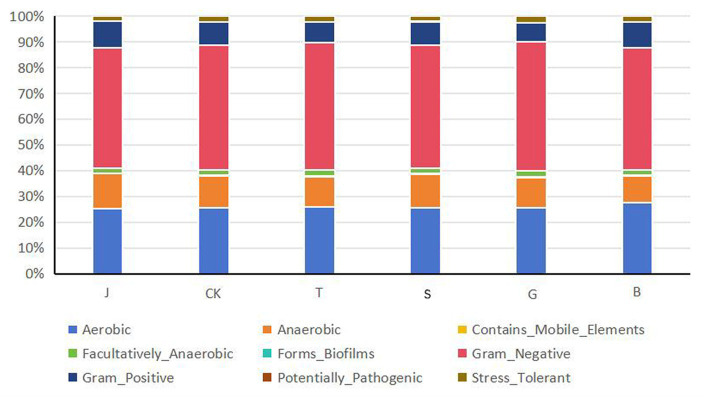
Prediction of bacterial BugBase phenotype under different treatments.

BugBase is a method for predicting the biological level coverage of functional pathways within complex microbiomes as well as biologically interpretable phenotypes (Ward et al., 2017). Following different drip irrigation treatments (Figure 8), the Aerobic bacteria in the soil changed compared to the control treatment CK, and the Aerobic bacteria decreased in J, S and G treatments, while the Aerobic bacteria increased in T and B treatments, and the T had the highest Aerobic bacteria abundance. While the abundance of Anaerobic bacteria in the soil of different treatments was opposite to that of the Aerobic bacteria, and the J had the highest number of Anaerobic bacterial species, and the B had the lowest number of species. The soil exhibited a higher level of Facultativeiy Anaerobic bacteria richness than the CK control group, and the relative abundance of said bacteria in the T was the most pronounced. The amount of mobile elements (Contains Mobile Elements) found in soil bacteria was significantly higher in the T and B treatments compared to the CK. The G exhibited slightly lower richness than the CK, whereas the J and S treatments showed lower rates than the CK; biofilm production by bacteria increases their chances of survival. Soils in the T and B treatments had the lowest number of bacteria with biofilm production (Forms Bioflims). However, bacteria with biofilm production were relatively more abundant in the J, S, and G treatments compared to the CK control treatment, with the highest abundance in the J. The proportion of J was the highest, and the corresponding bacteria exhibited a greater capacity to withstand external damage. Gram Positive peptidoglycan layer is thicker, while Gram Negative peptidoglycan layer is thinner (Wei, 2022). The relative abundance of Gram-Negative bacteria differed in various treatments, the relative abundance of J and B were very low, the relative abundance of J was slightly lower than the relative abundance of CK, and compared with the control group of CK, the relative abundance of T and G treatments had improved. The proportions of Gram-positive bacteria in soil were also different different treatments, and the corresponding treatments were opposite to the proportions of Gram-negative bacteria in soil, with the J treatment having the most Gram-positive bacteria in soil, and the G treatment having the least Gram-positive bacteria in the soil. After the maize crop underwent drip irrigation treatments at different growth stages, compared to the CK, the J, S and G treatments showed an increase in pathogenic (potentially pathogenic) bacteria, which was unfavorable for bacterial survival, while the T and B treatments showed a decrease in the relative abundance of pathogenic bacteria, and the B treatment had the lowest relative abundance, also had the highest survival rate. The J, S, and G treatments had fewer bacteria with oxidative Stress Tolerant, lower than the control group CK, the relative abundance of the T was slightly higher than that of the CK group, and the B treatment had the highest number of bacteria with oxidative Stress Tolerant in the soil, which was favorable for the survival of the bacteria. The differences between the different functional bacteria in the soil of the different treatments with increased drip irrigation were not significant compared to the control CK.

### 3.5 Phenotypic prediction of soil fungi FUNGuild

**Figure 9:**
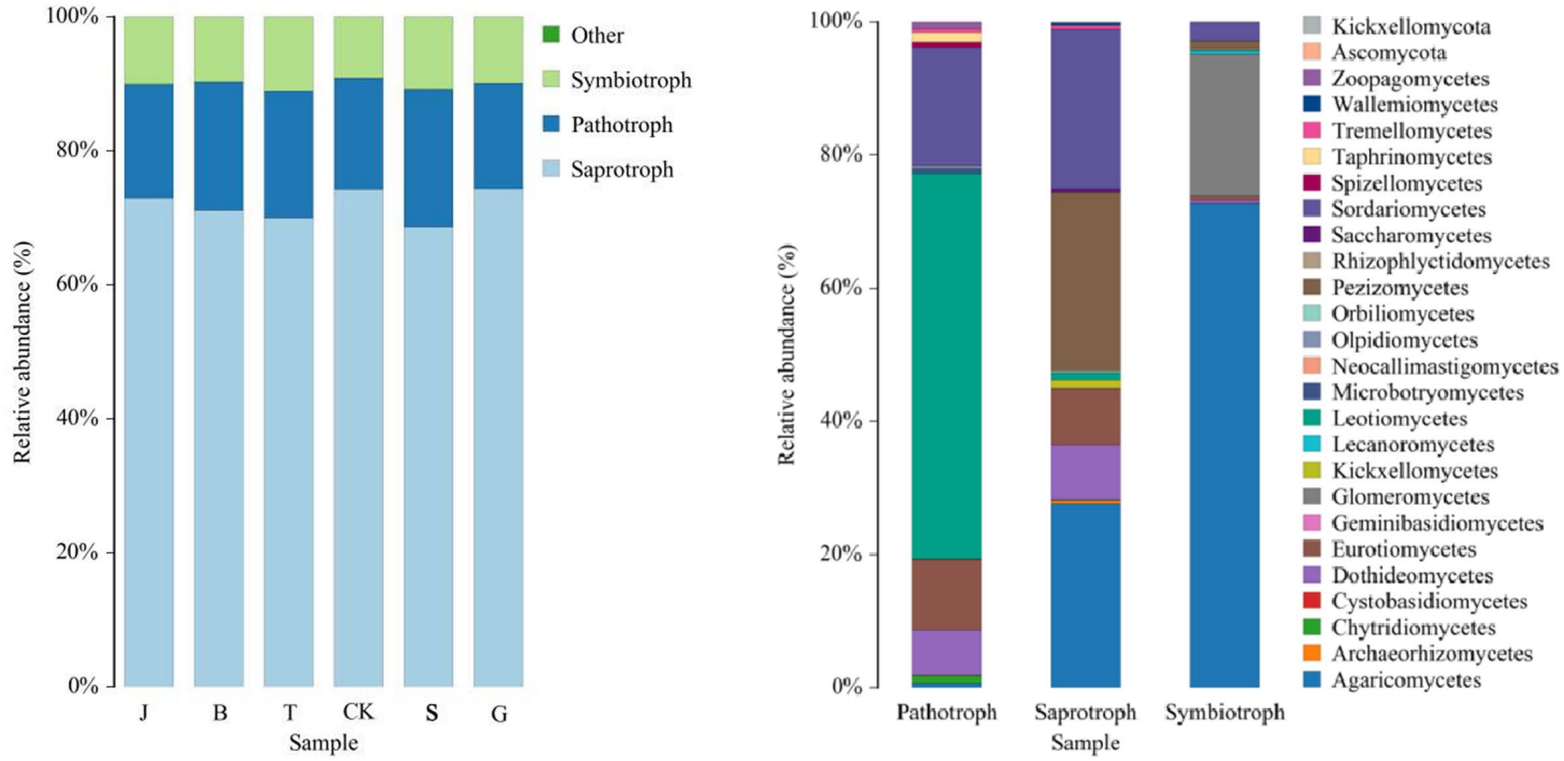
Prediction of FUNGuild phenotype of fungi under different treatments.

The prediction of the fungal community function in the soil after drip irrigation treatments of maize at different reproductive stages using FUNGuild revealed that the fungi were classified into three major groups according to the mode of nutrition (Figure 9), which were Saprotroph, Pathotroph, and Symbiotroph, with relative abundance percentages ranging from 68.60–74.33%, 15.76–20.60%, and 9.16–11.13%, respectively. The three main categories were further divided into 26 subcategories, and there were no significant differences in the functioning of the fungi in the soil after the different drip irrigation treatments compared to the control CK. The relative abundance of fungi in the pathotrophic group was highest in the Leotimycetes, followed by the Zoopagomycetes; in the saprotrophic group, Agarcomycetes and Pezizomycetes were the most abundant fungi; and in the symbiotroph group, the highest relative abundance was in the Dictyocarpaceae. In addition, Sordariomycetes, Eurotiomycetes, Orbiliomycetes, Saccharomycetes, Tremellomycetes, Chytridiomycetes and other fungi are present in the soil. Most fungi of the order Umbelliferae are symbiotic, a few are saprotrophic, and only a few are pathotrophic; the nutritional mode of Ascosphaera fungi is symbiotic.

## 4 Discussion

Soil microorganisms are an important component of soil, and their population size and activity can be used as an important index of soil quality and health (Zhong and Cai, 2004). The effects of increasing drip irrigation at different growth stages on soil microbial constituent groups and functions were different probably because of the differences in maize inter-root secretions during the different growth stages, which affect the soil microbial community structure (Bai et al., 2012), and the effects of soil water content on microbial respiration, as the microbial community structure is sensitive to changes in soil water content (Tian et al., 2018).

In this study, while focusing on the composition and structure of the soil microbial communities, we also investigated their functions. Functional prediction based on PICRUSt2 was performed on the bacterial community to reflect its functions, which can also indicate the level of soil fertilization to some extent. The primary functional prediction included six major biological metabolic pathways, such as “metabolism.” Metabolism accounted for as much as 77.45–78.24% of the total, playing a crucial role in the growth process of plants. The soil microbial community maintained relatively consistent metabolic functions across different drip irrigation treatments, which is consistent with the findings of Pang et al. (2021). Secondary function comparison using the KEGG database revealed 44 major secondary functions such as “carbohydrate metabolism” and “amino acid metabolism,” Carbohydrate metabolism, amino acid metabolism and energy metabolism are the basis of plant growth and development and play an important role in maintaining biochemical and metabolic processes (Pang et al., 2021). Carbohydrate metabolism involves the breakdown and conversion of carbohydrates into nutrients essential for plant growth (Yan et al., 2021), and amino acid metabolism involves the breakdown of proteins into ammonium nitrogen. The nutrient demands of maize vary at different growth stages, specifically for nitrogen, phosphorus, and potassium. Increasing drip irrigation can alter the availability of these nutrients in the soil (Wang, 2022), and the soil microbial community is affected by changes in these environmental factors (Zhu et al., 2023). This paper examines the impact of drip irrigation on the growth stages of crops and its effect on the prevalence of pathogenic bacteria. The study suggests that the decrease in nitrogen, phosphorus, and potassium content in the soil, particularly the significant reduction in available potassium content, may be responsible for the decrease in disease resistance of crops. Phenotypic prediction of soil bacterial function was performed based on BugBase. The J treatment had more anaerobic bacteria, the B treatment had more aerobic bacteria, which can decompose, absorb, and oxidize crop waste, and are beneficial for plant growth and development. The B treatment had bacteria with the lowest pathogenicity and the highest stress resistance. It has been confirmed that soil disease resistance is positively correlated with the relative abundance of Ascomycota and Actinomycota bacteri (Ai et al., 2015). Ascomycetes bacteria play an important role in promoting plant growth and improving soil ecosystems (Li et al., 2018). The growth of soil-borne pathogens can be effectively inhibited by actinobacteria (Huang et al., 2019). The functional prediction of the soil fungal community using FUNGuild found that saprotrophic, fungi were the most dominant trophic type of fungi in soil, and the relative abundance of Umbelliferae, which belong to the saprophytic type of fungi, was the highest in this study, which is in consistent with Chen XQ et al.’s findings (Chen et al., 2022). Saprotrophic bacteria decompose organic matter such as plant and animal residues, animal feces, and other organic matter in the soil (Li and Ma, 2018), which promotes crop uptake and has a positive effect on crop growth; however, pathotrophic bacteria increase the risk of plant diseases. If the number of bacteria and fungi in the soil is out of balance and the dominant flora is shifted, the chemical substances in the soil will change accordingly, and the stability of the soil ecosystem will gradually be reduced, affecting the normal growth of crops (Zhou and Yang, 2023).

## 5 Conclusion

Differences in soil microbial diversity indices between different growth stages of maize by increasing drip irrigation were not significant, with the highest number of soil bacteria at the large bell–tasseling stage and the highest number of soil fungi at the tasseling–filling stage. Community structure and functional diversity of soil bacteria and fungi were rich under the different irrigation regimes, and the primary functions of soil bacteria predicted by PICRUSt2 in the T, M, and B treatment groups as well as the secondary functions of soil bacteria in the B, G, and J treatment groups significantly differed from those in the CK group. It can be said that metabolism is the most basic and universal function of bacteria, and only some of them have strong genetic information processing, treatment of human diseases, environmental information processing and other functions. Furthermore, bacteria were functionally more diverse and more stable in number than fungi.

## Data availability statement

The raw data supporting the conclusions of this article will be made available by the authors, without undue reservation.

## Author contributions

LW: Conceptualization, Data curation, Investigation, Methodology, Software, Writing – original draft. XW: Funding acquisition, Project administration, Resources, Supervision, Validation, Writing – review & editing. TW: Investigation, Writing – review & editing.
